# Beyond Individual Features: Computational Analysis of Linguistic Coordination in Dementia Speech

**DOI:** 10.3390/brainsci16070763

**Published:** 2026-07-20

**Authors:** Vanessa Yoorhee, Gemma Moya-Galé

**Affiliations:** Teachers College, Columbia University, New York, NY 10027, USA; gm2446@tc.columbia.edu

**Keywords:** dementia, computational linguistics, computational analysis, linguistic features, DementiaBank, picture description task, inter-feature coordination, resource trade-off, natural language processing, speech analysis

## Abstract

**Highlights:**

**What are the main findings?**

- Dementia disrupts not only individual linguistic features but also the coordinative relationships between them; a preserved coordination linking syntactic simplification with lexical elaboration is present in healthy speakers but absent in people with dementia.
- Discourse-pragmatic factors, including self-referential language and hedging, moderate the relationships between linguistic features differently between the group with dementia and healthy speakers. This result suggests that the verbal output is shaped by discourse-level context which cannot be captured by the analysis of individual features.

**What are the implications of the main findings?**

- Inter-feature coordination may serve as a more sensitive marker of dementia-related language change than individual feature comparisons alone, with implications for computational modeling of dementia speech.
- The identified preserved coordination offers a longitudinally trackable target for speech–language pathologists monitoring language decline in dementia.

**Abstract:**

Background/Objectives. In dementia, progressive changes in spoken language, including reduced lexical diversity, increased disfluency, and syntactic simplification, are well documented. Although computational approaches have demonstrated reliable group differences in individual linguistic features, less attention has been given to the potential coordination between linguistic features and discourse-level moderation of the features with group differences. Methods. Over five domains—syntactic, lexical, disfluency, output, and affective—nine linguistic features were extracted from 552 transcripts (309 dementia, 243 healthy controls) of individuals performing the Cookie Theft picture description task in the DementiaBank Pitt Corpus. The Mann–Whitney U test was used to examine group differences with rank-biserial correlation effect sizes. Fisher z-transformation tests were applied to compare within-group correlation to examine the moderation effect of discourse-pragmatic factors (self-references, hedging, and sentiment polarity) on different groups. Fisher z-transformation tests were used to compare inter-feature correlations between the two groups. All multiple comparisons were controlled using the Benjamini–Hochberg false discovery rate (FDR) procedure. Results. Out of the nine features, word complexity, repetition rate, MLU, and filler rate were significantly different between the group with dementia and healthy controls. Among them, word complexity and repetition rate showed the largest and most consistent effects. Self-referential language showed a stronger association with verbal output in the group with dementia, although the association was not significant when analyses were restricted to one visit per participant. Moreover, one inter-feature relationship (i.e., the positive correlation between short utterance ratio and word complexity) remained significant after FDR correction. This correlation was present only in healthy controls, suggesting that this preserved coordination may be disrupted in dementia. Conclusions. Dementia disrupts not only individual linguistic features but also the relationships between them. The preserved coordination between syntactic simplification and lexical elaboration was found to be absent in speakers with dementia. Inter-feature coordination may, therefore, be able to reveal group differences that individual feature analyses alone cannot detect.

## 1. Introduction

### 1.1. Background

Dementia represents a significant challenge for global health. It is a progressive neurodegenerative disease affecting about 55 million people globally, and the number is predicted to triple by 2050 due to an aging global population [1]. Progressive memory loss and broader cognitive decline are often the defining features of dementia. However, changes in speech, such as reduced lexical diversity, increased disfluency, and less complex utterances, are one of the earliest and most measurable behavioral signs [2,3]. With the progression of dementia, these linguistic changes become more pronounced in connected speech, particularly in structured tasks such as picture description [4]. These impairments potentially extend beyond lexical and syntactic features to affective language, including prosody and emotional expression, which affects communication in daily life [5,6]. The analysis of spontaneous speech has become a compelling direction for the early and non-invasive detection of cognitive decline [7,8]. In particular, the Cookie Theft picture description task from the Boston Diagnostic Aphasia Examination [4] has been widely used as a standard elicitation method. This is because it produces connected and naturalistic speech in a structured context, which enables direct comparison of the language production between participants and healthy controls, as well as across participants.

These language changes reflect the decline in the cognitive system that supports language production [1,3]. As episodic and semantic memory are early systems disrupted in Alzheimer’s disease, lexical-semantic retrieval becomes more difficult, and anomia becomes evident with reduced lexical diversity and more frequent use of the limited range of words. According to Fraser et al. [2] and Klimova and Kuca [1], a bias toward the use of shorter and less specific vocabulary is one of the earliest markers of the disease.

Additionally, beyond lexical retrieval, coherent discourse requires planning, sequencing ideas, and connecting them across utterances, abilities that are impaired in speakers with dementia. Speakers with dementia who have impaired cognition gradually produce less informative and less cohesive discourse, with simpler grammar and shorter utterances [3,9]. These changes reflect the difficulty in connected speech, which increases as the disease progresses. People with dementia have been shown to produce narratives containing uncorrected errors and redundancies, consistent with the elevated repetition observed in this group [2,9]. These patterns suggest that the individual linguistic features that are different between individuals with dementia and healthy speakers may stem from a common underlying cognitive decline. Therefore, it is recommended to examine how these features relate to one another, rather than to treat them as individual and independent.

The goal of this study was to conduct a computational analysis of spontaneous speech in dementia. Here, computational analysis refers to the automated and quantitative extraction of linguistic features from speech transcripts, rather than manual coding or subjective clinical rating. This approach draws on natural language processing and statistical analysis. Computational analysis of dementia speech provides solid advantages over manual clinical assessment. First, it enables the systematic and reproducible extraction of linguistic features directly from transcriptions of speech. This can reduce reliance on subjective clinical judgment and enable comparison across large samples [2]. A recent systematic review of 51 studies with more than 17,000 participants confirmed that computationally derived linguistic features, including lexical diversity, syntactic complexity, and semantic coherence, are consistently strong predictors of cognitive impairment, and the combined approaches achieved diagnostic accuracies averaging 87% [10]. Previous studies with computational analysis have demonstrated reliable differences between groups with dementia and control groups in lexical, syntactic, disfluency, and affective domains [9,11,12,13]. Their findings emphasize the clinical relevance of computational speech analysis as a complement to neuropsychological assessment, as it has the potential to monitor communicative functions in dementia. The work of Fraser et al. [2] illustrates influential computational approaches. They applied machine learning classifiers and factor analysis to narrative speech data from DementiaBank Pitt Corpus, as the present study does, and they identified four latent factors: semantic impairment, acoustic abnormality, syntactic impairment, and information impairment. These factors reliably distinguished the group with dementia from healthy controls. Among these factors, semantic and syntactic factors were more strongly correlated in healthy speakers (r = 0.42) than in the group with dementia (r = 0.19). This result has an implication that these linguistic domains become functionally less integrated in dementia.

Previous computational studies have introduced machine learning classifiers, factor analysis, and automated feature extraction from transcripts. According to Shanker et al. [10], the lexical and syntactic features were the most discriminative factors, and disfluency and affective features showed a trend but required future validation. Collectively, these findings suggest that computational analysis in speech transcripts can provide reliable linguistic markers of dementia, which expands traditional neuropsychological assessment.

### 1.2. Gap

However, most previous studies have treated linguistic features as independent markers and have examined whether each feature differs between diagnostic groups [7,14]. This approach may overlook fundamental aspects of language impairment in dementia, in that communication breakdowns in dementia may represent coordination disruptions among different linguistic features. Dementia alters individual linguistic features; at the same time, it also disrupts the relationship between them, which is preserved in healthy speakers. Additionally, discourse-pragmatic factors, such as self-referential language, hedging, and sentiment polarity, have been underexplored in previous studies. Understanding the moderation effects of these factors on groups is meaningful for investigating how dementia affects language in naturalistic speech. To our knowledge, no prior study has investigated the coordination between linguistic features and the moderation effects of discourse-pragmatic factors simultaneously within the DementiaBank Pitt Corpus using a combination of computational methods.

### 1.3. Research Questions

This study investigates these gaps via computational analysis of nine linguistic features extracted from the DementiaBank Pitt Corpus [15], a public dataset consisting of 552 transcripts (309 with dementia and 243 healthy controls) of individuals performing the “Cookie Theft” picture description task. There are three research questions in this study. First, do speakers with dementia and healthy controls show significant differences in individual linguistic features (RQ1)? Second, do discourse-level factors, such as self-referential language, hedging, and sentiment polarity, exert a moderating effect on the relationship between linguistic features for each group distinctively (RQ2)? Third, do the pairwise relationships between linguistic features show differences between the group with dementia and healthy speakers? If so, which pairs show the most divergence (RQ3)?

## 2. Materials and Methods

This study used a publicly available deidentified corpus; no additional data were collected in the present study, and no ethical approval was required beyond the original corpus developers [15].

### 2.1. Participants and Data

A publicly available corpus from TalkBank, the DementiaBank Pitt Corpus [15], was used. The Pitt Corpus is composed of participants referred from the Benedum Geriatric Center at the University of Pittsburgh Medical Center and recruited through local medical networks. Participants were required to be over 44 years of age and with at least 7 years of education, no history of nervous system disorders, and an initial Mini Mental State Examination (MMSE) score of 10 or greater. The diagnosis of dementia was based on the history of cognitive and functional decline as determined by the results of neuropsychological and mental status examinations [15]. From this corpus, the transcripts of participants performing the Cookie Theft picture description task [4] were included. During the task, participants were asked to describe a scene that shows a kitchen with several given circumstances. The Cookie Theft task has been widely used in dementia research studies. The corpus includes 552 transcripts from 292 unique participants, as some individuals contributed more than one transcript across longitudinal visits (addressed in Section 2.4). Of these transcripts, 309 were from people with dementia (mean age = 71.3 years, SD = 8.7; 117 male, 189 female, 3 unspecified) and 243 were from healthy controls (mean age = 64.2 years, SD = 8.0; 89 male, 154 female). The speech data were provided in a .cha format file [16]. The group with dementia is composed of individuals with probable or possible Alzheimer’s disease as well as other diagnoses [15]. As the corpus was collected longitudinally, MMSE scores in the sample are spread in a wider range than the initial enrollment threshold (dementia: M = 19.7, SD = 5.6, range 1–30; control: M = 29.1, SD = 1.1, range 24–30). To maintain a single, consistent elicitation task and diagnostic protocol, the dataset was restricted to the Pitt Corpus. A secondary corpus considered, ADReSS, was excluded because participant overlap with the Pitt Corpus was identified. The years of education of the participants had a mean of 12.7 years (SD = 3.0) in the group with dementia and 14.1 years (SD = 2.4) in controls.

### 2.2. Transcript Processing

Each transcript had a structure that interleaves speaker tiers. Participant speech was marked with the *PAR tier, and investigator speech was indicated by the *INV tier. Only *PAR utterances were analyzed, and *INV speech was excluded. During preprocessing, CHAT format annotations, nonverbal markers, and transcription codes were removed with custom parsing functions to prepare clean utterances for analysis. All analyses of transcripts were conducted in Python, and a random seed was fixed as 42 for reproducibility across the pipeline.

### 2.3. Linguistic Features

Linguistic features extracted from cleaned participant utterances were classified across five domains: syntactic complexity, lexical characteristics, disfluency, output, and affective language. These five domains were selected because they are relevant to language impairment in dementia and the feasibility for automatic extraction from transcripts, which is consistent with computational approaches to dementia speech analysis [2,7]. Discourse-level features, such as coherence in narration, coreference tracking, and propositional density, were not included in this study because of the constrained nature of the Cookie Theft picture description task, which limits the range of naturally elicited discourse behaviors [4]. Discourse-level analysis is reserved for future studies, with expansion of the sample including conversational and narrative speech samples. Similarly, semantic features were also excluded, as the fixed visual stimulus in the task limits semantic variability and reduces the possibility of discrimination of semantic measures across participants. Even though complex discourse-level features were excluded due to the constrained nature of the task, simpler discourse-related variables, such as self-references, hedging, and sentiment polarity, were included as moderators to detect contextual variation in language use. Self-reference rate was operationalized following Tausczik and Pennebaker [17], hedging frequency following Hyland [18], and sentiment polarity using TextBlob 0.19.0, a Python library that provides rule-based sentiment and subjectivity scores, following De Smedt and Daelemans [19]. Prior to analysis, features were screened for multicollinearity using Spearman correlations. Unique word count, total word count, and total verb count were eliminated due to high intercorrelation (r > 0.89). The final set of linguistic features consisted of nine measures across five domains, as summarized in Table 1. Self-reference rate was computed as the proportion of first-person singular pronouns or determiners, such as “I,” “my,” “me,” “mine,” and “myself,” relative to total words. Hedging frequency is computed as the proportion of hedging terms, such as “maybe,” “probably,” “perhaps,” “might,” and “seem,” relative to total words.

For syntactic features, mean length of utterance (MLU) and short utterance ratio were included. MLU, which is defined as the mean number of words per utterance, has been widely used in connected speech to measure grammatical complexity [3]. Short utterance ratio, defined as the proportion of utterances containing three words or fewer, represents a measure of syntactic fragmentation, as people with dementia produce fewer complex syntactic units in picture description tasks [20]. Lexical features were represented by type–token ratio (TTR) and word complexity. TTR, which is the ratio of unique words to total words, is widely used to measure lexical diversity and has been shown to decline in dementia [11]. Word complexity, which is defined as the mean number of syllables per word, was used to measure lexical sophistication, as linguistic ability and vocabulary richness in early life are linked to cognitive function and dementia later in life [12]. Disfluency features were composed of repetition rate and filler rate. Repetition rate is the proportion of repeated word n-grams, sequences of n consecutive words. It is included because people with dementia repeat words and phrases more frequently than control groups in picture description tasks [9]. Filler rate, that is, the proportion of filled pauses, such as “uh,” “um,” relative to total words, was included because filled pauses play a distinct role in spontaneous speech [21] and may reflect word retrieval difficulties in dementia. To measure output features, utterance count and informativeness were included. Utterance count means overall verbal output volume per transcript, and informativeness is the proportion of content words relative to total words. They serve as a representation of propositional content efficiency. Both have been used to identify language decline in spontaneous speech in dementia [22,23]. Finally, in this study, subjectivity was included as an exploratory measure of affective expression in connected speech because emotional processing and affective language are altered in dementia [5,6]. It is defined as the mean TextBlob subjectivity score per utterance, which ranges from 0 (fully objective) to 1 (fully subjective).

### 2.4. Statistical Analysis

Statistical analyses were conducted in Python 3.12.13 (Python Software Foundation, Beaverton, OR, USA) using the SciPy 1.16.3 and statsmodels 0.14.6 libraries. Shapiro–Wilk tests evaluated the distribution of all features prior to the analysis. A nonparametric approach was applied, as most linguistic features showed non-normality.

#### 2.4.1. Group Differences

Mann–Whitney U tests were applied to analyze nine linguistic features between the two groups, people with dementia (*n* = 309) and healthy controls (*n* = 243), in the Pitt Corpus (N = 552). To calculate effect sizes, rank-biserial correlation (r_rb), which is appropriate for effect size measurements for Mann–Whitney U tests, was used, because r_rb does not assume normality, unlike Cohen’s d. The interpretation of effect sizes followed conventional standards: negligible (|r| < 0.10), small (|r| ≥ 0.10), medium (|r| ≥ 0.30), and large (|r| ≥ 0.50) [24]. Positive r values represent higher values in the group with dementia relative to controls, and negative values indicate higher values in the control group. A post hoc sensitivity analysis indicated that, with 309 participants with dementia and 243 control participants, this study had 80% power (α = 0.05, two-tailed) to detect effects as small as d ≈ 0.24, which indicates adequate sensitivity for the small-to-medium effects observed.

#### 2.4.2. Moderation Analysis

To examine whether discourse factors moderate group differences, within-group Spearman correlations were computed for each pair of a linguistic feature and a moderator separately in the group with dementia and healthy controls. Then, the two correlations were compared with Fisher z-transformation tests. This investigated whether the association between a linguistic feature and a moderator differed by group. Three moderators—self-reference rate (Self_Refs), hedging frequency (Hedges), and sentiment polarity (Polarity)—were examined against seven of the nine linguistic features (MLU, utterance count, TTR, word complexity, repetition rate, filler rate, and subjectivity), and this yielded 21 feature–moderator comparisons. The Benjamini–Hochberg false discovery rate procedure was applied for all 21 tests, as this rank-based approach is less sensitive to extreme values, such as skewed and zero-inflated moderators, which can distort ordinary least squares regression estimates.

#### 2.4.3. Three-Way Relationships

To examine whether the relationships between linguistic features differed by diagnostic group—which may reflect different organization of linguistic systems in dementia—pairwise feature associations were analyzed in each group, and their strength was compared. Spearman correlations were computed for all pairwise combinations of the nine features separately for the dementia and control groups. Spearman correlations were selected over Pearson correlations due to their robustness to outliers and non-normal distributions. Correlation coefficients were compared between the two groups using Fisher z-transformation tests, which is the standard method for testing whether two coefficients differ significantly. In testing a total of 36 feature pairs (C(9,2) = 36), multiple comparisons were controlled with the FDR procedure. Results that passed FDR correction are reported as confirmed findings; results that are significant at raw *p* < 0.05 without surviving FDR are reported as exploratory.

The DementiaBank Pitt Corpus includes repeated, longitudinal recordings from some participants. The corpus is composed of 552 transcripts from 292 unique individuals (1.9 transcripts per participant on average). Because of this non-independence of the data, two robustness analyses were conducted. First, a single-visit analysis included only one transcript per participant (*n* = 292). Second, linear mixed-effects models with a random intercept for each participant were fitted, which retained all transcripts while accounting for within-participant correlation. Findings that held across these analyses are described as robust; these are referred to below as the single-visit subset and the mixed-effects model.

## 3. Results

### 3.1. Group Differences in Linguistic Features (RQ1)

**Figure 1:**
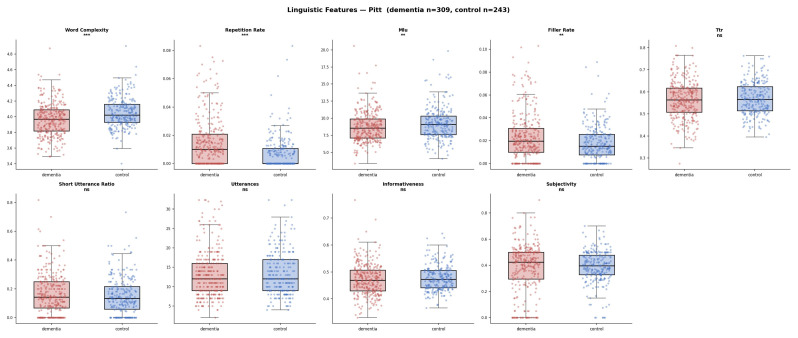
Distributions of the nine linguistic features for the group with dementia (*n* = 309) and control (*n* = 243) groups in the Pitt Corpus. Boxes show medians and interquartile ranges with individual observations overlaid; significance markers reflect Mann–Whitney U tests (** *p* < 0.01, *** *p* < 0.001; ns = not significant).

**Figure 2:**
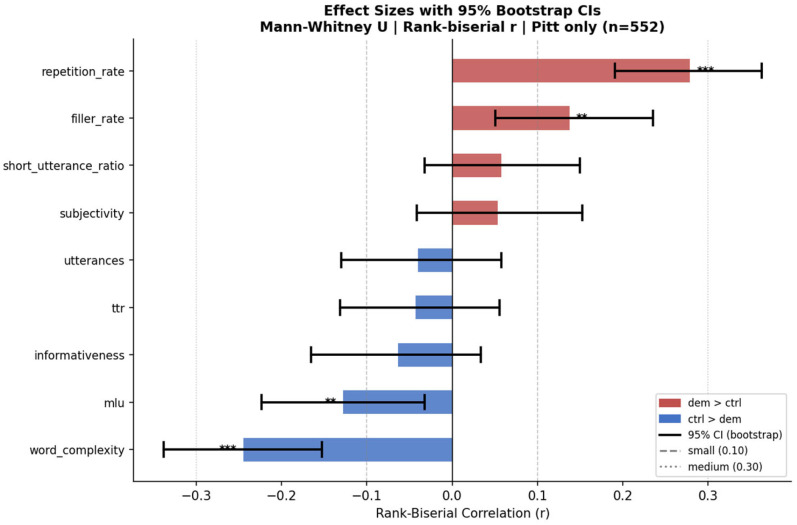
Group-difference effect sizes (rank-biserial correlation r) for the nine linguistic features, with 95% bootstrap confidence intervals. Positive values indicate higher scores in the group with dementia and negative values higher scores in controls; dashed and dotted reference lines mark the small (0.10) and medium (0.30) effect-size thresholds, respectively. ** (*p* < 0.01) and *** (*p* < 0.001).

Four of the nine linguistic features differed significantly between the group with dementia and healthy controls (Table 2; Figure 1 and Figure 2). The speech from the group with dementia showed lower word complexity than the control group, one of the two largest group differences observed (Mdn 3.97 vs. 4.02; r = −0.24). The speakers with dementia repeated words and phrases more frequently (Mdn 0.010 vs. 0.000; r = +0.28) and produced more fillers (Mdn 0.020 vs. 0.015; r = +0.14), with a lower mean length of utterance (Mdn 8.58 vs. 9.08; r = −0.13). Word complexity and repetition rate showed the most robust differences, with bootstrap confidence intervals excluding zero. The distributions are visualized in Figure 1.

The remaining five features, type–token ratio, short utterance ratio, utterance count, informativeness, and subjectivity, did not differ significantly between groups (Table 2).

### 3.2. Moderation Effects (RQ2)

**Figure 3:**
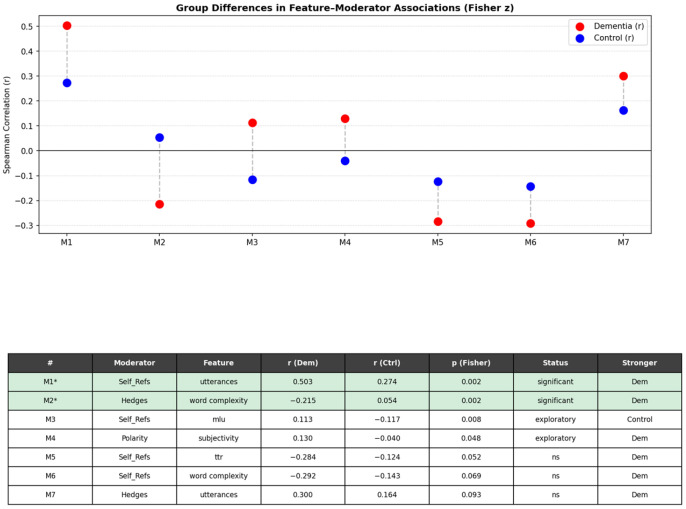
Group differences in feature–moderator associations. For each feature–moderator pair, within-group Spearman correlations were computed separately in the dementia and control groups and compared using Fisher z-transformation tests (Benjamini–Hochberg FDR correction across all 21 comparisons). The seven pairs with the greatest group divergence are shown (M1–M7); M1 (self-references × utterance count) and M2 (hedging × word complexity) remained significant after FDR correction. ‘*’ marks pairs surviving FDR correction and ‘ns’ denotes non-significant; ‘#’ is the column header for the row index (M1–M7).

To investigate the moderation effect by discourse context, within-group Spearman correlations between each linguistic feature and each moderator were compared across groups using Fisher z-transformation tests. FDR correction was applied to all 21 feature–moderator comparisons (Figure 3). After the FDR correction, two comparisons remained significant: hedging × word complexity and self-references × utterance count. Hedging and word complexity were associated in opposite directions for the two groups. The relationship was negative in the group with dementia (r = −0.22) while absent in controls (r = +0.05; p_FDR = 0.017). This pattern was consistent when the analysis was restricted to one visit per participant. Self-referential language showed a stronger association with utterance count in the group with dementia (r = 0.50) than in the control group (r = 0.27; p_FDR = 0.017), which was attenuated and treated as preliminary because it was not significant in the single-visit subset. These results suggest that discourse-pragmatic factors affect linguistic output differently in dementia.

### 3.3. Feature Interrelationships by Diagnostic Group (RQ3)

**Figure 4:**
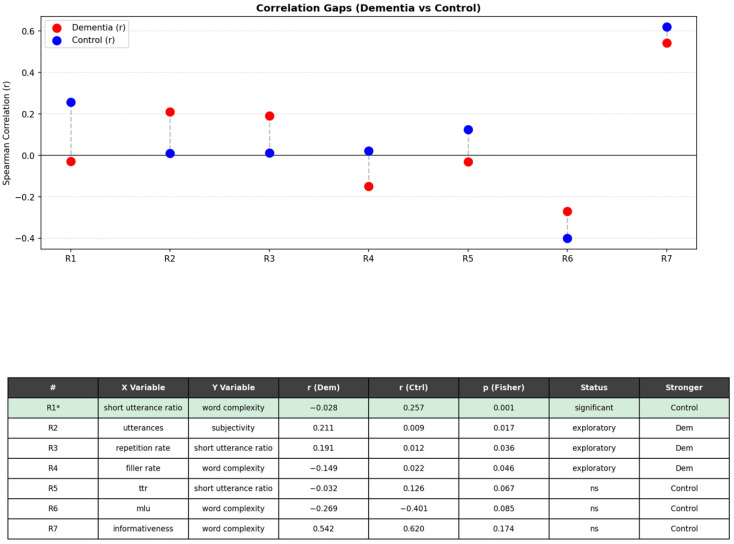
Differences in pairwise feature associations between groups. Upper panel: Spearman correlations for the dementia and control groups across the seven most divergent feature pairs (R1–R7). Lower panel: Corresponding Fisher z test results; the short utterance ratio × word complexity pair (R1) remained significant after FDR correction. ‘*’ marks the pair surviving FDR correction and ‘ns’ denotes non-significant; ‘#’ is the column header for the row index (R1–R7).

Pairwise feature correlations were compared between the dementia and control groups using Fisher z tests, with FDR correction applied to all 36 *p*-values. Pairs that remained significant after FDR correction were classified as confirmed findings; pairs significant at the uncorrected threshold (*p* < 0.05) were classified as exploratory. The results are included in Figure 4. One pair remained significant after FDR correction. Short utterance ratio and word complexity appeared to have different relationships in different groups (dementia: r = −0.028; control: r = +0.257; Fisher z *p* = 0.0008). In the control group, speakers who produced shorter utterances tended to use words with more syllables. However, this relationship was not observed in the group with dementia. Although only one pair passed FDR correction, three additional pairs were also significant at an uncorrected threshold. These are reported as exploratory findings pending replication. Utterances and subjectivity showed a contrasting pattern (dementia: r = +0.210; control: r = +0.009; *p* = 0.018), and greater verbal output was associated with higher subjectivity only in the group with dementia. Repetition rate and short utterance ratio showed a positive association in the group with dementia that was absent in controls (dementia: r = +0.191; control: r = +0.012; *p* = 0.036). Filler rate and word complexity were negatively associated in the group with dementia but not in controls (dementia: r = −0.149; control: r = +0.022; *p* = 0.045). The remaining 29 pairs were non-significant at the uncorrected threshold and are not reported individually. Among all the four significant pairs (one FDR confirmed, three exploratory), word complexity and short utterance ratio each appeared in two of the four significant pairs, which suggests that these two features are the most central to group-differentiated linguistic coordination.

## 4. Discussion

In this study, linguistic feature patterns were examined in the spontaneous speech from a picture description task performed by speakers with dementia and controls. The data were extracted from the Pitt Corpus (*n* = 552), and the analysis involved nine features across five domains. Three research questions were presented: group differences in individual features (RQ1), moderation of group effects (RQ2) by discourse-level moderators, and inter-feature relationship differences by group membership (RQ3). The primary finding of this study is that dementia disrupts not only individual features but also the relationships between them. A preserved coordination, in particular, links a short utterance ratio with word complexity, which is present only in the control groups and not in speakers with dementia.

### 4.1. Group Differences in Individual Features (RQ1)

After the Mann–Whitney U tests, four features appeared as significant in terms of group differences: word complexity, repetition rate, MLU, and filler rate. Word complexity and repetition rate had the strongest and most consistent evidence. Both have bootstrap confidence intervals that do not cross zero, and MLU and filler rate, although smaller in magnitude, also remained significant after FDR correction (p_FDR = 0.022 and 0.016, respectively). Despite small effect sizes, this result is consistent with prior work using comparable corpora and methods with automated extraction that can introduce noise reducing effect size [2,7,10]. The group with dementia produced words of lower complexity than the control groups, which replicates the well-documented pattern of lexical simplification in dementia speech. This is consistent with previous reports of reduced lexical richness and word complexity in the speech of people with dementia [11]. Fraser et al. [2] also showed that semantic impairments lead to shorter and less specific words in narratives produced by speakers with dementia. Similarly, Klimova and Kuca [1] characterized lexical retrieval difficulty, i.e., anomia, circumlocution, and substitution of generic terms, as the earliest and most consistent language markers in dementia. The reduced word complexity in the present study is consistent with this pattern: as lexical retrieval becomes compromised, speakers with dementia increasingly rely on shorter, less complex words in place of longer, multisyllabic alternatives (word frequency itself was not measured in the present study). In speakers with dementia, the repetition rate was elevated, as expected. These findings replicate those from Tomoeda et al. [9] and Fraser et al. [2], who identified content repetition as a distinguishing feature of dementia narratives in the Cookie Theft task. Repetition in dementia is often understood as a consequence of degraded episodic monitoring, the inability to track one’s own speech, rather than a primary linguistic impairment [2]. The effect is small but reliable, and with word complexity, it represents the most robustly supported finding of RQ1. The MLU of speakers with dementia was modestly lower than that of the control groups, which is consistent with Kemper et al.’s [3] longitudinal findings showing syntactic simplification in dementia speech. Beltrami et al. [8] observed reduced utterance length in early dementia even when surface grammatical correctness was largely preserved. Filler rate was higher in speakers with dementia than in controls, in line with Clark and Fox Tree [21], who distinguished filled pauses as markers of lexical retrieval difficulty; although this difference was attenuated in the single-visit subset, it remained significant in the mixed-effects model. Beltrami et al.’s [8] study also showed that acoustic and temporal disruptions that include pause-related features are sensitive markers of cognitive decline. Still, both effects are smaller in magnitude than those of word complexity and repetition rate; filler rate was attenuated in the single-visit subset but remained significant in the mixed-effects model. The remaining features that are not significantly different by groups are TTR, short utterance ratio, utterance count, informativeness, and subjectivity. The non-significance of TTR replicates a recurring finding, as lexical diversity measures are sensitive to transcript length and show inconsistent group differences in picture description tasks, where both groups produce relatively limited output [2,11]. On the other hand, the non-significance of informativeness is more surprising, as prior studies have consistently found reduced informativeness in speakers with dementia [22,23]. This disparity possibly reflects the limitations of automated feature extraction for this measure, unlike previous studies that used manual annotation of information. These non-significant individual findings do not reduce the importance of these features, because features that do not differentiate the groups individually participate in meaningful inter-feature relationships that differentiate the groups.

### 4.2. Moderation by Discourse-Level Moderators (RQ2)

The association between self-referential language and utterance count was significantly stronger in the group with dementia than in controls (Fisher z, p_FDR = 0.017), although this association was attenuated when analyses were restricted to one visit per participant and should be interpreted with caution. When the speakers with dementia used self-referential language, such as “I,” “my,” “me,” “mine,” and “myself,” the utterance counts became higher. However, this association was weaker and non-significant in controls. This suggests that speakers with dementia who rely more on first-person anchoring also tend to produce more utterances. This pattern could reflect an adaptive strategy, in which first-person anchoring helps maintain discourse when word retrieval is difficult, or it also could be a perseverative tendency that speakers with dementia return repeatedly to self-referential language without necessarily intending to. This finding is consistent with previous research on pronoun use in dementia. According to Fraser et al. [2], the pronoun-to-noun ratio was elevated with semantic impairment, which reflects a shift to more generic and self-oriented language. However, this interpretation needs more future work showing whether this pattern reflects an intentional communication strategy that remains preserved in dementia or a consequence of degraded content tracking. The remaining 19 moderation comparisons were not significant after FDR correction. The non-significance of sentiment polarity in moderation implies that, in the constrained picture description context, the emotional tone of the language does not substantially alter the group differences in core linguistic features. Similarly, subjectivity as a standalone feature or as a moderator may have greater sensitivity in natural discourse speech, which is worth exploring in future studies using narrative or conversational speech data.

A second moderation also differed significantly across groups and remained significant in the single-visit subset: hedging was associated with word complexity in opposite ways across groups. In dementia, more frequent hedging was associated with simpler vocabulary (r = −0.22), whereas in controls, the two were unrelated (r = +0.05). This suggests that in dementia, hedging and reduced word complexity may surface together as signs of word-finding difficulty—a combination absent in healthy speakers.

### 4.3. Differential Inter-Feature Relationships (RQ3)

This result can be interpreted as a preserved coordination, or resource trade-off, grounded in a cognitive resource-allocation framework, in which syntactic planning and lexical retrieval draw on the same limited resources, such as working memory and executive function [3]. In neurotypical language production, constructing grammatically complex sentences requires more resources for syntactic planning than for lexical retrieval, so simpler words are used; conversely, shorter utterances free resources for greater lexical precision. This trade-off—exchanging syntactic simplification for lexical elaboration—implies preserved executive control over a flexible allocation of resources, consistent with prior work on language production under cognitive load [3] and on cognitive reserve and linguistic compensation [12]. In dementia, neurodegeneration affecting the frontal and temporal regions disrupts this allocation, so this coordination breaks down. Although individual features also differ between groups (RQ1; [2]), the disruption of their coordination reflects an additional, network-level impairment beyond changes in any single feature.

This finding is aligned with Fraser et al.’s [2] factor analysis showing semantic and syntactic impairment factors that were more correlated in controls than in groups with dementia. That is, in healthy speakers, these domains are functionally linked, whereas they become disrupted in dementia. The present study extends this logic from correlation between broad factors to correlation between specific linguistic features that are computationally defined. The present study, therefore, provides a finer-grained and statistically explicit test of feature coordination using Fisher z-transformation tests, yielding results that are directly replicable with other feature sets.

There are three additional feature pairs that appeared as nominally significant group differences but did not survive FDR correction. They are, therefore, treated as exploratory. The group with dementia showed a positive association between utterance count and subjectivity (r = +0.210), and this association was absent in the control group. In the group with dementia, speakers who produced more output also tended to express more subjective or emotionally toned content, which suggests that greater verbal output may be accompanied by more affective language in this group. Another positive association between repetition rate and short utterance ratio in dementia was also absent in controls. This may indicate that the fragmentation of utterances occurs with repetition as a disruption in speech. The negative association between filler rate and word complexity (r = −0.149) was also absent in controls, as anticipated. This suggests that disfluency and lexical simplification may also co-occur in dementia speech. Altogether, these exploratory patterns suggest that the relationships of linguistic features, particularly those involving word complexity and short utterance ratio, may carry discriminative information that individual comparisons alone cannot capture; however, replication in independent samples, and particularly in more naturalistic or conversational speech tasks, is needed to confirm this interpretation, as the constrained picture description task may limit the range of group differences.

### 4.4. Theoretical Implications

These findings support the view that dementia-related language impairment involves a network-level disruption rather than isolated deficits. The most distinctive characteristic of dementia speech may be the loss of coordination between linguistic features, such as the breakdown of the coordinated relationships that characterize speech production in healthy individuals. This perspective is consistent with the broader computational linguistics literature that emphasizes the importance of feature interactions and inter-domain relationships in distinguishing dementia from control speech [2,10], though previous studies have not directly tested pairwise correlation differences in linguistic features between groups using the approach employed in this study.

The perspective about the connectivity between linguistic domains is consistent with neuroscientific evidence that dementia is reflected not only in regional impairment but also in disrupted connectivity between brain regions that are responsible for linguistic performance [2]. In the present study, all of the four significant feature pairs crossed domains. This implies that cross-domain coordination may be vulnerable to dementia-related disruption.

Even though subjectivity was not significant as a group difference marker individually, this feature appeared in one of the four nominally significant exploratory pairs, specifically, the association with utterance count observed only in the group with dementia. Future work examining the affective dimension of speech in dementia is warranted. Gong et al. [13] demonstrated that emotional variability in speech distinguished speakers with Frontotemporal Dementia (FTD) from healthy controls even when emotional status did not differ. This is a conceptual parallel to the finding of this study that feature relationships including subjectivity may reveal group differences that individual feature comparisons alone cannot detect. According to Sola et al. [6], prosodic emotion recognition was selectively impaired in dementia, which implies that affective features of speech may be more sensitive to pathological change when examined dynamically rather than as static group means. Future studies combining subjectivity scores with prosodic measures that are extracted from audio may strengthen the affective dimension of computational analysis in speech. For instance, pitch variability and speech rate are potentially strong factors that may reflect the affective dimension of computational dementia speech analysis. This multimodal approach will enable the examination of the generalizability of the relational patterns beyond transcript-based features to acoustic and paralinguistic dimensions of speech.

### 4.5. Limitations

First, this study relied on a single corpus, the Pitt Corpus, with the setting of a single task, the Cookie Theft picture description task. The task is widely used and well validated [2,4] but limits the range of observable linguistic behaviors. A more extensive dataset would be helpful to evaluate the generalizability of the inter-feature relationships identified in this study in other discourse contexts. Second, because all features were extracted from transcripts rather than audio recordings, acoustic and prosodic dimensions of speech, such as pitch, timing, and pause patterns, could not be examined.

In particular, a tool developed for written text, TextBlob, was used to compute features such as subjectivity and polarity. However, the original purpose of this tool is not for clinical speech analysis, which may introduce measurement error when analyzing spontaneous dementia speech. Therefore, further validation of these automated tools against manual annotation specifically in dementia speech corpora is recommended to strengthen the validity of these measures as indicators of affective and pragmatic language use.

Third, the sample is cross-sectional, not longitudinal. This means that the preserved coordination identified in RQ3 cannot be interpreted as a within-person change over time. Future work is needed to examine whether the coordination between short utterance ratio and word complexity changes over time in individual speakers, which can develop a longitudinal framework as Gkoumas et al. [14] did.

Fourth, all reported group differences and inter-group correlation differences were significant. It is true that the group with dementia and the healthy controls were significantly different in their age (Mann–Whitney U, *p* < 0.001). However, they remained significant after controlling for age using partial Spearman correlations computed on all available age data, *n* = 459. This means that age does not account for the reported effects. Education level and MMSE were not included as covariates because both are closely confounded with diagnosis in the Pitt Corpus. Their contributions should be examined in future work [7].

Fifth, the three discourse-level moderators—self-references, hedges, and polarity—were measured using automated tools that are developed for general text. Therefore, their validity as proxies for discourse-level function in dementia speech should be examined in future studies. In addition, participants’ race, ethnicity, and information about the first language are not reported in the Pitt Corpus. This can limit generalizability across linguistically and culturally diverse populations. In this study, MLU is defined as utterance length. Incorporating finer-grained, parse-based structural syntactic measures would be a useful direction for future studies [3,25,26,27].

Finally, the sample of this study, 552 transcripts from 292 unique individuals, includes longitudinal visits of several participants, so it is possible that the observations are not completely independent. To investigate the impact of this structure, a single visit per participant (*n* = 292) was examined. The results were consistent for word complexity and repetition rate. Word complexity was significantly lower in the group with dementia than in healthy speakers, and the repetition rate was significantly higher in the group with dementia. Short utterance ratio × word complexity, which reflects the preserved coordination, also remained significant. In healthy speakers, word complexity increased when utterances became shorter, and the pattern was absent in people with dementia. The hedging × word complexity moderation was also consistent across the robustness analyses: more frequent use of hedging words (“maybe,” “probably”) was associated with simpler vocabulary in people with dementia, whereas the two were unrelated in healthy controls. This may suggest that in the group with dementia, hedging and reduced word complexity represent their word-finding difficulty. However, filler rate, which was significant with all transcripts, no longer reached the threshold with one visit per person. In both groups, people who used more self-referential words tended to speak more, and this link was stronger in the group with dementia. However, the gap between the two groups was no longer statistically significant when each person was counted only once. To confirm all observations while accounting for this structure, linear mixed-effects models with a random intercept for each participant were also fitted. These confirmed the primary findings: word complexity, repetition rate, MLU, and filler rate all differed significantly between groups (all *p* < 0.05), and both the short utterance-ratio × word-complexity interaction (*p* < 0.001) and the hedging × word complexity moderation (*p* = 0.009) remained significant, whereas the self-reference × utterance count moderation did not (*p* = 0.75). Notably, filler rate, which was attenuated in the single-visit subset, remained significant in the mixed-effects model.

## 5. Conclusions

This study examined nine linguistic features across five domains in the spontaneous speech from 552 transcripts (292 unique participants) in the DementiaBank Pitt Corpus. It examined group differences in individual features, discourse-level moderation, and inter-feature relationships across groups through computational analysis. Whereas the differences in individual features observed here largely replicate previous findings, the primary contribution of this study is the analysis of coordination between features: beyond differences in individual features, dementia also disrupted the coordination among them. A preserved coordination links a short utterance ratio with word complexity. However, in the group with dementia, this coordination was absent; in healthy controls, r = +0.257, and in speakers with dementia, r = −0.028. This pairwise coordination was the only inter-feature relationship that remained significant after FDR correction, which indicates that this coordination between syntactic and lexical processes is selectively disrupted by dementia. Two discourse-pragmatic moderations also remained significant after FDR correction. Hedging was associated with word complexity differently across groups, and self-referential language was more strongly associated with verbal output in dementia, although the latter was attenuated when analyses were restricted to one visit per participant. These results indicate that discourse-pragmatic factors influence linguistic output differently across groups in ways that individual feature analyses cannot detect. Taken together, while these findings should be regarded as preliminary, they are consistent with the hypothesis that clinically relevant information is reflected in the relationships between linguistic features, in addition to the individual features themselves. These results have meaningful implications in both computational modeling and clinical practice. For researchers, the findings suggest that pairwise feature coordination may offer discriminative signals beyond what the comparisons of individual features can provide. Further development of feature engineering pipelines for dementia detection may help clarify these signals. For speech–language pathologists, the identified preserved coordination that is disrupted in dementia offers a target to monitor longitudinally. For instance, tracking whether the coordination between syntactic simplification and lexical elaboration changes in individual speakers in the long term may yield clinically meaningful information about disease progression. Therefore, future work should extend this framework to longitudinal approaches with extended corpora and conversational speech contexts to establish the generalizability of inter-feature coordination as a marker of cognitive decline.

## Figures and Tables

**Table 1:** Linguistic Features.

Domain	Features
Syntactic	MLU, short utterance ratio
Lexical	TTR, word complexity
Disfluency	Repetition rate, filler rate
Output	Utterance count, informativeness
Affective	Subjectivity

*Note.* MLU = mean length of utterance; TTR = type–token ratio.

**Table 2:** Group differences in linguistic features across the Pitt Corpus (N = 552). Note. Mdn = median; r_rb = rank-biserial correlation (effect size); Sig = significance level. Effect size thresholds following [24]: small r ≥ 0.10, medium r ≥ 0.30. Informativeness medians are not reported from this dataset due to variable availability. Sig reflects Benjamini–Hochberg FDR-corrected significance.

Feature	Domain	Dem Mdn	Ctrl Mdn	r_rb	*p*	Sig
Word complexity	Lexical	3.97	4.02	−0.244	<0.001	***
Repetition rate	Disfluency	0.010	0.000	+0.279	<0.001	***
MLU	Syntactic	8.58	9.08	−0.128	0.010	*
Filler rate	Disfluency	0.020	0.015	+0.137	0.005	*
TTR	Lexical	0.564	0.571	−0.043	0.388	ns
Short utterance ratio	Syntactic	0.143	0.133	+0.057	0.244	ns
Utterances	Output	12.00	12.00	+0.040	0.417	ns
Informativeness	Output	NA	NA	−0.064	0.198	ns
Subjectivity	Affective	0.425	0.397	+0.054	0.280	ns

*** *p* < 0.001; * *p* < 0.05; ns = not significant; NA = not available.

## Data Availability

The data presented in this study are from the DementiaBank Pitt Corpus. The dataset is a publicly available resource accessible at https://dementia.talkbank.org (accessed on 19 March 2026), with approval. The nine extracted linguistic features per participant and the analysis code supporting this study are available from the corresponding author on reasonable request.
